# Upper Limb Strength and Function Changes during a One-Year Follow-Up in Non-Ambulant Patients with Duchenne Muscular Dystrophy: An Observational Multicenter Trial

**DOI:** 10.1371/journal.pone.0113999

**Published:** 2015-02-02

**Authors:** Andreea Mihaela Seferian, Amélie Moraux, Mélanie Annoussamy, Aurélie Canal, Valérie Decostre, Oumar Diebate, Anne-Gaëlle Le Moing, Teresa Gidaro, Nicolas Deconinck, Frauke Van Parys, Wendy Vereecke, Sylvia Wittevrongel, Michèle Mayer, Kim Maincent, Isabelle Desguerre, Christine Thémar-Noël, Jean-Marie Cuisset, Vincent Tiffreau, Severine Denis, Virginie Jousten, Susana Quijano-Roy, Thomas Voit, Jean-Yves Hogrel, Laurent Servais

**Affiliations:** 1 Institute of Myology, Paris, France; 2 Department of Child Neurology, Groupe de Recherches sur l’Analyse Multimodale de la Fonction Cérébrale, Centre Hospitalier Universitaire d’Amiens, Amiens, France; 3 Department of Pediatrics, Division of Pediatric Neurology and Metabolism, Neuromuscular Reference Center, Universitair Ziekenhuis Gent, Gent, Belgium; 4 Department of Child Neurology, Assistance Publique—Hôpitaux de Paris Hôpital Trousseau, Paris, France; 5 Department of Child Neurology, Assistance Publique—Hôpitaux de Paris Hôpital Necker Enfants Malades, Paris, France; 6 Department of Child Neurology, Centre Hospitalier Régional Universitaire de Lille—Hôpital Roger-Salengro, Lille, France; 7 Department of Physical Medicine and Rehabilitation, Centre Hospitalier Régional Universitaire de Lille—Hopital Pierre Swynghedauw, Lille, France; 8 Reference Center for Neuromuscular Disease, Centre hospitalier régional de la Citadelle, Liège, Belgium; 9 Department of Pediatrics, Centre de références Maladies Neuromusculaires Garches-Necker-Mondor-Hendaye, Université de Versailles Saint-Quentin-en-Yvelines Assistance Publique—Hôpitaux de Paris Hôpital Raymond Poincaré, Garches, France; 10 Thérapie des maladies du muscle strié / Institut de Myologie, Unité Mixte de Recherche S 974 Université Pierre et Marie Curie—Institut national de la santé et de la recherche médicale—Formation de Recherche en Evolution 3617 Centre national de la recherche scientifique—Association Institut de Myologie, Paris, France; The Hospital for Sick Children, CANADA

## Abstract

**Introduction:**

Upper limb evaluation of patients with Duchenne Muscular Dystrophy is crucially important to evaluations of efficacy of new treatments in non-ambulant patients. In patients who have lost ambulation, there are few validated and informative outcome measures. In addition, longitudinal data demonstrating sensitivity to clinical evolution of outcome measures over short-term periods are lacking.

**Patients and Methods:**

We report here the results of a one-year multicenter study using specifically designed tools to assess grip, pinch strength, and hand function in wheelchair-bound patients. Our study assessed 53 non-ambulant patients with Duchenne muscular dystrophy aged 17.1 ± 4.8 years (range: 9 – 28.1 years). The average Brooke functional score of these patients was 4.6 ± 1.1. The average forced vital capacity was 44.5% predicted and 19 patients used non-invasive ventilation. Patients were assessed at baseline, 6 months, and one year using the Motor Function Measure and innovative devices (namely the MyoSet composed of MyoGrip, MyoPinch, and MoviPlate).

**Results:**

Our study confirmed preliminary data previously reported regarding feasibility of use and of reliability of the MyoSet and the correlation at baseline between distal strength and clinical outcomes such as FVC, Brooke score, age, and duration since loss of ambulation. A significant correlation was observed between the distal upper limb strength and clinical variables. The sensitive dynamometers (MyoGrip and MyoPinch) and MoviPlate captured a 12-month change in non-ambulant Duchenne muscular dystrophy patients of all ages.

**Trial Registration:**

ClinicalTrials.gov NCT00993161 NCT00993161

## Introduction

Out-of-frame deletions in the dystrophin gene usually lead to Duchenne muscular dystrophy (DMD). Patients with DMD become symptomatic before the age of 5 years and generally lose ambulation before the age of 15. The evolution of the disease leads to impaired mobility and subsequent loss of function not only in lower but also in upper limbs [1, 2]. Encouraging results in clinical evaluation of novel approaches for treatment of DMD [3–5] has increased the number of clinical trials in the recent years [6]. Most ongoing therapeutic trials focus on young ambulant DMD patients, and appropriate outcome measures have been developed to monitor effects of therapy on these patients. The preferred primary outcome for DMD evaluation is the 6-minute walk distance [7, 8]. Less can be said about the patients who are drawing near the full-time wheelchair use or those who have lost ambulation. The 2007 International Workshop on Outcome Measures [9] stressed the importance of finding the appropriate outcome measures for non-ambulant patients. For these patients, identifying measures to cover the distal movements (fingers) is of great importance [10].

Observer-rated performance-based scales are currently used as assessment tools for upper limbs in DMD patients. A recent critical review of the existing tools for upper limbs done by Mazzone *et al*. shows that functional ability in the daily life of these individuals is incompletely addressed by Motor Function Measure (MFM), Jebsen Hand Function Test, or Upper Limb Functional Ability Test [11]. Self-reported measures like Egen Klassifikation, ABILHAND (questionnaire for manual ability), and ACTIVLIM (scale of activity limitations) cover a larger spectrum of activities not observed during clinical evaluation. Mayhew *et al*. developed a new module—Performance of the Upper Limb—specifically designed for DMD patients to assess upper limb function in ambulant and non-ambulant patients starting from the age of 5 [12, 13]. The weakness of the scales lies in the evaluator subjectivity and limited possible scores (1 point score system on a maximum of 7 points).

A novel approach for upper limbs evaluation was recently proposed by Kurillo *et al*. [14, 15] using a stereo camera-based reachable-workspace analysis system. This system was able to distinguish individuals with varying degrees of proximal upper limb functional impairments. A pilot study using the Kinect platform to measure functional reaching volume and movement velocity showed encouraging results in a small DMD cohort [16]. This approach is of great interest for patients with preserved proximal mobility, but lack of sensitivity for distal movements limits its utility.

Dynamometers have been used in strength evaluation in patients with various diseases including DMD [17, 18]. Correlations between strength and function have been described in DMD patients [19–21]. Strength measures with the quantitative muscle testing (QMT) system have been used in natural history studies [22, 23] and clinical trials [24, 25]. MyoPinch, MyoGrip, and MoviPlate are tools recently developed at the Institute of Myology to assess upper limb strength and function in neuromuscular patients. We demonstrated the feasibility and reliability of these tools in tests of 30 non-ambulant DMD patients and 30 age-matched male controls [26]. This manuscript describes a study using the same tools and presents the baseline data for all 53 (30 previously reported and 23 presented here for the first time) non-ambulant DMD patients included in the study. We have assessed the sensitivity to change of MyoPinch, MyoGrip, and MoviPlate measurements over one year and have determined the sample size of DMD non-ambulant patients needed in clinical trials to prove whether a given drug effectively stabilizes the disease in non-ambulant patients.

## Patients and Methods

The protocol for this trial and supporting CONSORT checklist are available as supporting information; see S1 TREND Checklist and S2 Protocol.

### Patients

The present study is part of a multicenter observational study for upper limb evaluation in non-ambulant patients with neuromuscular disorder (ClinicalTrials.gov Identifier: NCT00993161) that took place between January 2010 and January 2013 and present results of DMD patients only. The study design and the preliminary results at baseline for the first 30 DMD patients were previously reported [26]. Patients from different neuromuscular centers from France (Institute of Myology, Trousseau Hospital, Necker Hospital, Paris; Raymond Poincaré Hospital, Garches; University Hospital, Lille) and Belgium (University Hospital, Gent; CHR La Citadelle, Liege) were invited to participate in the study. Functional and strength assessments were carried out at each recruiting center. All data from medical histories (age of loss of ambulation, spine deformities, cardiac and respiratory evaluation, intellectual disability, corticosteroids intake) were reviewed. IQ was not formally tested for all patients, but when available, was considered as normal if above 80 [27]. The study was approved by the French Ethics Review Board Paris VI and the Belgian Ethics Review Board of Gent and Liège. Before inclusion, all patients or their parental authorities provided signed informed consent.

Patient inclusion criteria for the multicenter observational study were: age 8 to 30 years, genetically confirmed neuromuscular disorder, and complete loss of ambulation defined as inability to walk 10 meters in the standard conditions of the 6-Minute Walk Test [28]. Exclusion criteria were major cognitive impairment, inability to stay seated for one hour, recent upper limb surgery or trauma, or treatment by corticosteroids initiated less than 6 months prior to the start date of the study. In the analysis reported here, only data on patients with genetically confirmed DMD were included.

### Testing devices

Distal upper limb strength and function were assessed for the dominant and the non-dominant sides for each patient using the MyoSet composed of MyoGrip and MyoPinch devices (strength) and the MoviPlate device (motor ability) as described [26]. When performing the MoviPlate, three different positions are noted: 1) forearm in pronation and in contact with the plate; 2) the maneuver is done with one or more fingers touching the cylindrical targets and the forearm is almost not touching the plate; 3) the patient supports his cubital side on the plate and touches the cylinders with a finger and the forearm is in contact with the plate.

### Protocol

All patients were evaluated at baseline and 6 and 12 months later. The clinical data recorded at every visit included most recent respiratory data (e.g., forced vital capacity (FVC), type of ventilation), cardiac data (e.g., left ventricular ejection fraction), orthopedic and functional status (including Brooke scale), and the disease history. Baseline assessment encompassed a test-retest evaluation. Both arms were tested using the MyoSet with the side randomly chosen for the first assessment. All patients were given between two and five tests (depending whether last score was lower and reproducible within 10% of previous trials) and the maximal value was recorded on each side. The MFM was also performed at baseline and 6 and 12 months later. Since the patients were all non-ambulant, the total score and D2 (axial and proximal motor skills) and D3 (distal motor skills) sub-scores were used for analysis. Details of the experimental protocol were previously reported [26]. For this paper, we chose to present data only for MyoSet and MFM; the tapping test and the MyoWrist described by Servais *et al*. were not used in the present analysis due to lacking data in some patients.

### Statistical Analyses

**Correlations at baseline of strength, function, and clinical parameters**. Correlations between strength values (MyoGrip, MyoPinch) and functional scores (MoviPlate, MFM scores) at baseline on both dominant and non-dominant sides were determined using a non-parametric Spearman correlation coefficient. Correlation between baseline strength values (MyoGrip, MyoPinch) and functional scores (MoviPlate, MFM scores) on both dominant and non-dominant sides with clinical parameters (age, height, weight, duration since loss of ambulation, FVC, Brooke score) at baseline was also analyzed using a non-parametric Spearman correlation coefficient, given the obvious nonlinear relation between values. Baseline values corresponded to the maximum value reached of both test and retest sessions.

### One-year follow up

One-year follow up was assessed by performing a repeated measure ANOVA on strength values and functional scores using side and visit as factors. Only patients who were tested at baseline, 6 months and 1 year were included. We first assess the normality of the variables using a test of Shapiro-Wilk. It appeared that MovPlate and MFM value were normally distributed, whilst MyoGrip, MyoPinch, and MFM sub-scores were not. Therefore, we used non-parametric Wilcoxon test to compare variables. Comparisons were verified using Student test for normal values. If a visit effect was found, one-year scores were compared with baseline score using non parametric Wilcoxon test on all available data (including subjects who were not evaluated at a 6-months visit).

In order to assess whether the change over one year was dependent on the disease stage, one-year differences were tested for correlation with the duration since loss of ambulation at baseline using a non-parametric Spearman correlation coefficient to take into account possible nonlinear relationships. If a significant correlation was found, scores as a function of duration since loss of ambulation were graphically assessed and cut-offs visually estimated. Visit effects and one-year differences were then reassessed in each subgroup as previously explained. The same analyses were performed on the MFM total score and MFM sub-scores.

The sample size for a future randomized clinical trial with two independent groups was assessed using the sample size formula described previously [29]. The difference to detect was chosen as a stabilization of strength or motor function in the treated group compared to the natural evolution of strength and motor function in the placebo group; this was estimated based on natural history data collected in this study. The standard deviation was calculated as the standard deviation of the one-year differences from the natural history group. The alpha risk was set at 5% and the power at 80%. The analysis was performed on data from patients of all ages and on the subgroups of patients with duration since loss of ambulation under or more than 3 years.

All analyses were performed using the SPSS 19 statistical software (SPSS Inc., Chicago, IL). The limit of statistical significance was set to 0.05.

## Results

**Figure 1:**
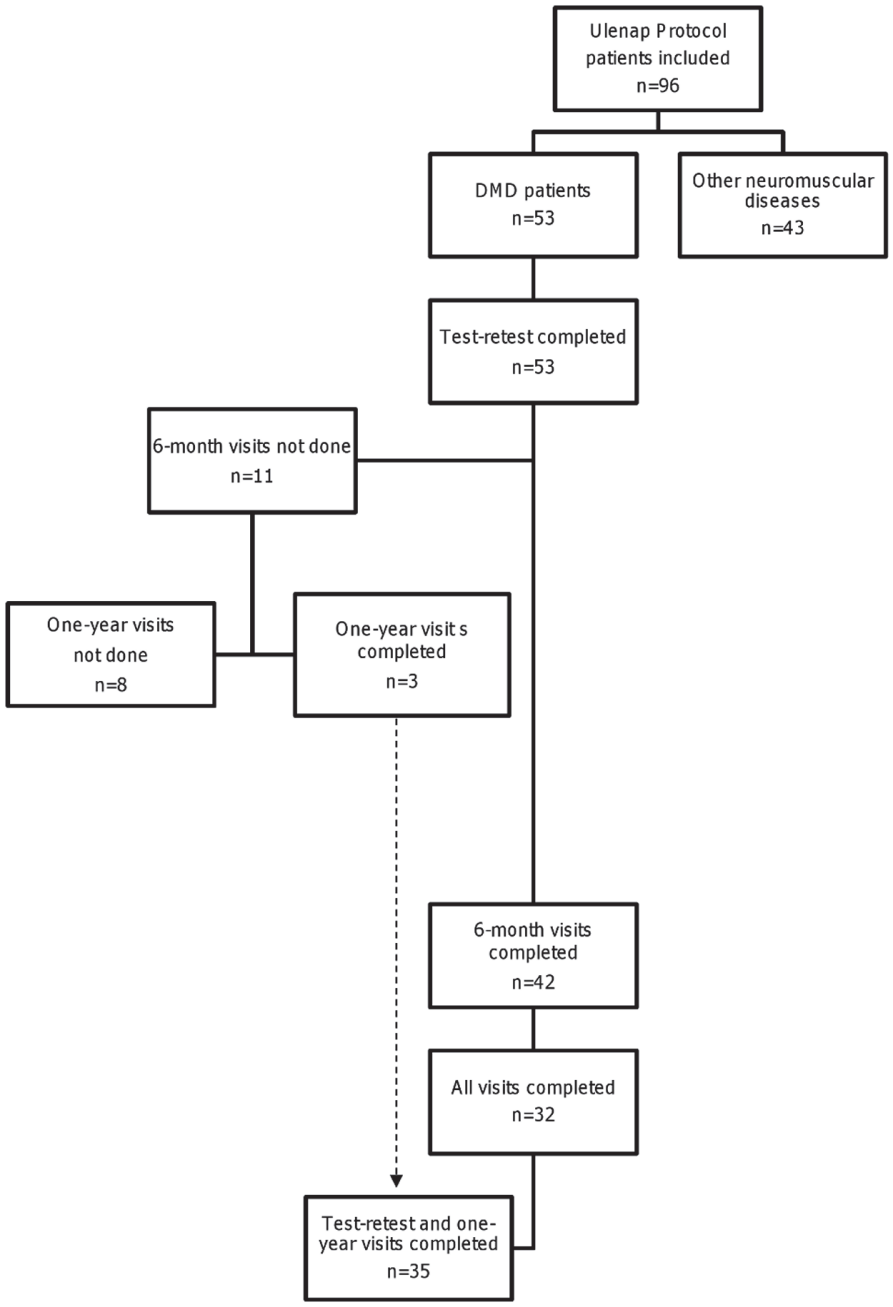
Flow chart of patients included in the clinical protocol.

Fifty-three DMD patients were initially included in the study and 35 completed the one-year evaluation (Fig. 1). The clinical features of patients are presented in Tables 1 and 2. Eighteen patients prematurely left the study: Five patients refused to continue (three patients after the 6-month visit); four were lost to sight; one underwent surgery; one was included in another study; one missed his last visit; and the others left the study for unknown reasons.

**Table 1:** Clinical data of all DMD patients sorted by age.

	**Age (years)**	**Mutation**	**H**	**W**	**CS**	**LOA**	**SS**	**ID**	**LVEF**	**FVC**	**Brooke (score#)**
§	9.0	del45–52	124	34	no	96	no	no	64	60	3
*	10.0	c.7392delC	140	29.5	no	84	no	yes	71	104	4
§	10.2	del21	135	21	no	105	no	no	65	56	4
	10.6	del8–13	135	43	yes	120	no	no	62	77	2
* §	10.8	del53	134	32.5	yes	122	no	yes	64	90	2
* §	11.6	c.998C>A	151	34.6	no	116	no	yes	64	58	5
§	11.9	dup8–9	155	68	yes	108	no	no	NA	55	5
*	12.1	del3–44	161	39	no	118	no	no	NA	43	5
* §	12.2	dup8–11	158	55	yes	108	no	yes	61	65	4
	12.3	del48–54	NA	34	yes	144	no	no	NA	NA	2
§	12.8	c.9459_9462del	156	48	no	120	no	yes	68	60	4
* §	12.9	del46–49	140	33.5	no	123	yes	yes	55	48	5
	13.1	del48–50	162	50	no	126	no	yes	66	68	5
* §	13.7	del48–54	162	74	no	123	yes	yes	57	26	3
* §	13.8	del31–43	176	90	no	132	no	no	50	57	3
* §	13.8	c.10453dup	160	54	no	150	no	no	66	101	2
*	14.2	dup2–5	154	47	no	108	no	no	35	63	5
§	14.2	del5–7	162	46.7	no	114	no	no	71	61	5
* §	14.4	c. 7657C>T	155	28.5	no	118	yes	yes	64	41	5
§	14.4	del24–43	165	38.7	no	84	yes	no	55	47	NA
* §	15.2	del8–9	171	52	no	99	no	no	60	64	5
* §	15.5	c.998C>A	166	40	no	169	yes	no	52	40	5
* §	15.7	del42–54	155	29	no	156	yes	yes	86	33	4
* §	15.7	del10–11	150	29.8	no	120	yes	yes	65	30	5
*	15.9	del45	166	32	no	119	no	no	50	47	5
*	16.0	c.4870C>T	ND	58	no	106	yes	no	35	63	5
§	16.5	del45–50	157	55.8	no	156	yes	yes	45	25	5
* §	16.6	c.4084C>T	175	68	no	99	yes	no	65	50	5
	17.1	del3–24	150	42	no	84	yes	yes	56	8	6
§	17.6	del46–47	160	49	no	168	yes	no	72	65	4
* §	17.7	c.6364G>T	158	23	no	91	yes	no	71	12	5
§	17.8	dup56–63	165	63	no	120	yes	yes	NA	11.1	5
*	17.8	c.7858dup	164	42.5	yes	135	yes	no	62	39	5
§	17.9	del5–7	152	70	no	156	yes	no	74	61	5
*	18.1	del45–57	170	50	no	135	yes	yes	62	37	5
* §	18.2	del49–50	160	58	no	96	yes	no	60	17	5
* §	18.8	c.10722delC	147	32.3	no	NA	yes	yes	49	NA	5
§	19.5	del32	160	31.8	no	168	yes	no	NA	26	5
*	19.5	c.2638delC	171	67	no	118	yes	no	68	18	5
	19.6	c.3772_3776delins	175	94	no	NA	no	no	41	90	2
* §	20.1	del51–60	NA	89.5	no	133	yes	yes	60	49	6
§	20.4	c.2947C>T	170	83	no	108	yes	yes	45	33	5
	20.5	del48–50	157	47	yes	123	no	no	61	33	5
* §	22.0	del8–43	172	46	no	125	yes	no	35	14	5
	22.2	c.4779delTins37	155	44	no	96	yes	no	76	NA	6
* §	22.4	del47–51	160	50	no	102	yes	yes	59	23	6
§	23.5	del10–11	166	59	yes	132	yes	no	52	27	5
*	23.9	del42–44	173	71	no	126	yes	no	50	26.3	5
	24.0	dup44	170	47	no	144	yes	no	56	17	5
* §	26.7	del51	162	73	no	132	yes	no	45	14	5
* §	27.7	del 45–54	163	48	no	144	no	no	50	12	5
	27.7	c.10567G>T	172	66	no	108	no	no	59	16	5
§	28.1	del7–10	171	69.6	no	96	yes	no	60	NA	6

* patients previously published.

§ patients who accomplished the 12-month visits.

CS—corticosteroids use; H—height (cm), W—weight (kg), FVC—forced vital capacity (% of predicted value), ID—intellectual disability, LOA—age at loss of ambulation (months), LVEF—left ventricular ejection fraction (%), NA—not available, SS- spine surgery.

**Table 2:** Clinical and functional data at baseline.

	**N**	**Mean (SD)**	**Median [Min-Max]**
Age (years)	53	17.1 (4.8)	16.5 [9.0–28.1]
Weight (kg)	53	50.6 (17.7)	48 [21–94]
Height (cm)	50	159 (11.7)	160 [124–176]
Age at loss of ambulation (months)	51	121.2 (21.9)	120 [84–169]
Time spent in wheelchair (years)	51	6.87 (5)	5.7 [0.3–20.1]
Corticoids (number of patients)	53		8
Ventricular ejection fraction (%)	48	58.7 (10.9)	60 [35–86]
Forced vital capacity (% of normal values)	49	44.5 (24.3)	43 [8–104]
Brooke score (#)	53	4.6	5 [2–6]
Spine surgery (number of patients)	52		31
MFM-D2 (#)	53	41.6 (26.6)	38.9 [0–94.4]
MFM-D3 (#)	53	70.5 (21.9)	76.2 [9.5–95.2]
MFM-Total (#)	53	31.7 (14.8)	32.3 [2.1–62.5]

### Clinical features at baseline

Of the patients initially enrolled, none were invasively ventilated, but 16 used night non-invasive ventilation and three used non-invasive ventilation continuously. Fourteen patients had left ventricular ejection fractions lower than 55%, and five of these patients had dilated cardiomyopathy. Genetic analyses of the initially enrolled subjects revealed 32 exonic deletions, five duplications, and 16 point mutations among the subjects.

### Clinical features at one-year

Of the patients who completed the one-year follow up, eleven patients used night non-invasive ventilation (the other five did not participate at the last visit). Nine patients had a left ventricular ejection fraction inferior to 55% (the other five did not participate at the last visit) and 4 of them had dilated cardiomyopathy. Spine surgery had been performed on 31 patients at a mean age of 14 years. Mild intellectual disability was noted in 35.8% of all the patients. The mutations of the patients who completed the one-year visit were comprised of 23 deletions, three duplications and nine point mutations.

### Feasibility

Due to major joint retractions, one patient could not perform all the tests with his non-dominant hand; one patient could not perform at all the MyoGrip and the MoviPlate; and two other patients could not perform at all the MoviPlate. MyoPinch was not performed in one patient on both sides at retest because subject tiredness. MyoGrip was not performed on one patient at inclusion due to a lack of availability of the equipment. The weakest patient scored 0.05 kg for grip and 0.07 kg for pinch.

### Baseline reliability

We first examined the reliability of our parameters. All the measurements showed very high intra-rater reliability according to intraclass correlation coefficient (ICC) values (all > 0.9). Reliability was not statistically different in patients with intellectual disability compared to patients without intellectual disability for any of the tests.

### Baseline correlation between strength and function (MyoGrip or MyoPinch versus MoviPlate or MFM)

The grip and pinch strengths positively correlated with the MoviPlate or the MFM functional tests (rho > 0.5, with the exception of the correlation between MyoPinch and MoviPlate, which was slightly lower with rho ranging from 0.4 to 0.5) (Table 3).

**Table 3:** Correlations between strength and functional tests at baseline for the dominant (D) and the non-dominant (ND) sides.

	**MoviPlate**		**MFM-D2 (%)**		**MFM-D3 (%)**		**MFM-Total (%)**	
	**N**	**rho**	**N**	**rho**	**N**	**rho**	**N**	**rho**
MyoGrip-ND (kg)	48	0.51 **	50	0.77 **	50	0.86 **	50	0.82 **
MyoGrip-D (kg)	48	0.60 **	51	0.78 **	51	0.87 **	51	0.82 **
MyoPinch- ND (kg)	49	0.39 **	53	0.77 **	53	0.82 **	53	0.81 **
MyoPinch- D (kg)	49	0.48 **	53	0.81 **	53	0.86 **	53	0.84 **

*p-value<0.05, **p-value<0.01.

### Baseline correlation between MyoSet and clinical parameters

**Figure 2:**
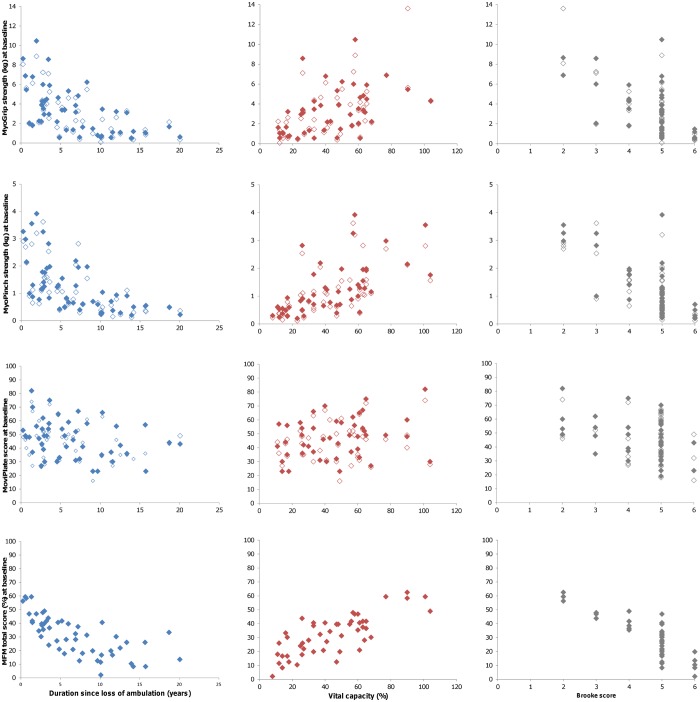
Correlation between MyoSet values at baseline and clinical data.

All tests were negatively correlated with the Brooke score. Data from all tests, with the exception of MoviPlate on the non-dominant hand, were positively correlated with FVC and negatively correlated with age and duration since loss of ambulation (Table 4). There was no significant correlation between test scores and weight or height (data not shown). Data on correlations are summarized graphically in Fig. 2.

**Table 4:** Correlation between the MyoSet and MFM at baseline on dominant (D) and non-dominant (ND) sides with clinical and motor function parameters.

	**Age (years)**		**Duration since loss of ambulation (years)**		**FVC (%)**		**Brooke (#)**	
	**N**	**rho**	**N**	**rho**	**N**	**rho**	**N**	**rho**
MyoGrip-ND (kg)	50	-0.48 **	48	-0,63 **	46	0.50 **	45	-0.62 **
MyoGrip-D (kg)	51	-0.49 **	49	-0,63 **	47	0.55 **	46	-0.59 **
MyoPinch-ND (kg)	53	-0.58 **	51	-0,71 **	49	0.69 **	48	-0.67 **
MyoPinch-D (kg)	53	-0.58 **	51	-0,70 **	49	0.70 **	48	-0.70 **
MoviPlate-ND (#)	49	-0.16	47	-0,25	45	0.19	44	-0.23
MoviPlate-D (#)	49	-0.15	47	-0,26	46	0.25	44	-0.35 *
MFM-D2 (#)	53	-0.65 **	51	-0,73 **	49	0.70 **	48	-0.76 **
MFM-D3 (#)	53	-0.55 **	51	-0,66 **	49	0.62 **	48	-0.68 **
MFM-Total (#)	53	-0.67 **	51	-0,76 **	49	0.72 **	48	-0.78 **

*p-value<0.05, **p-value<0.01.

### One-year follow-up

**Figure 3:**
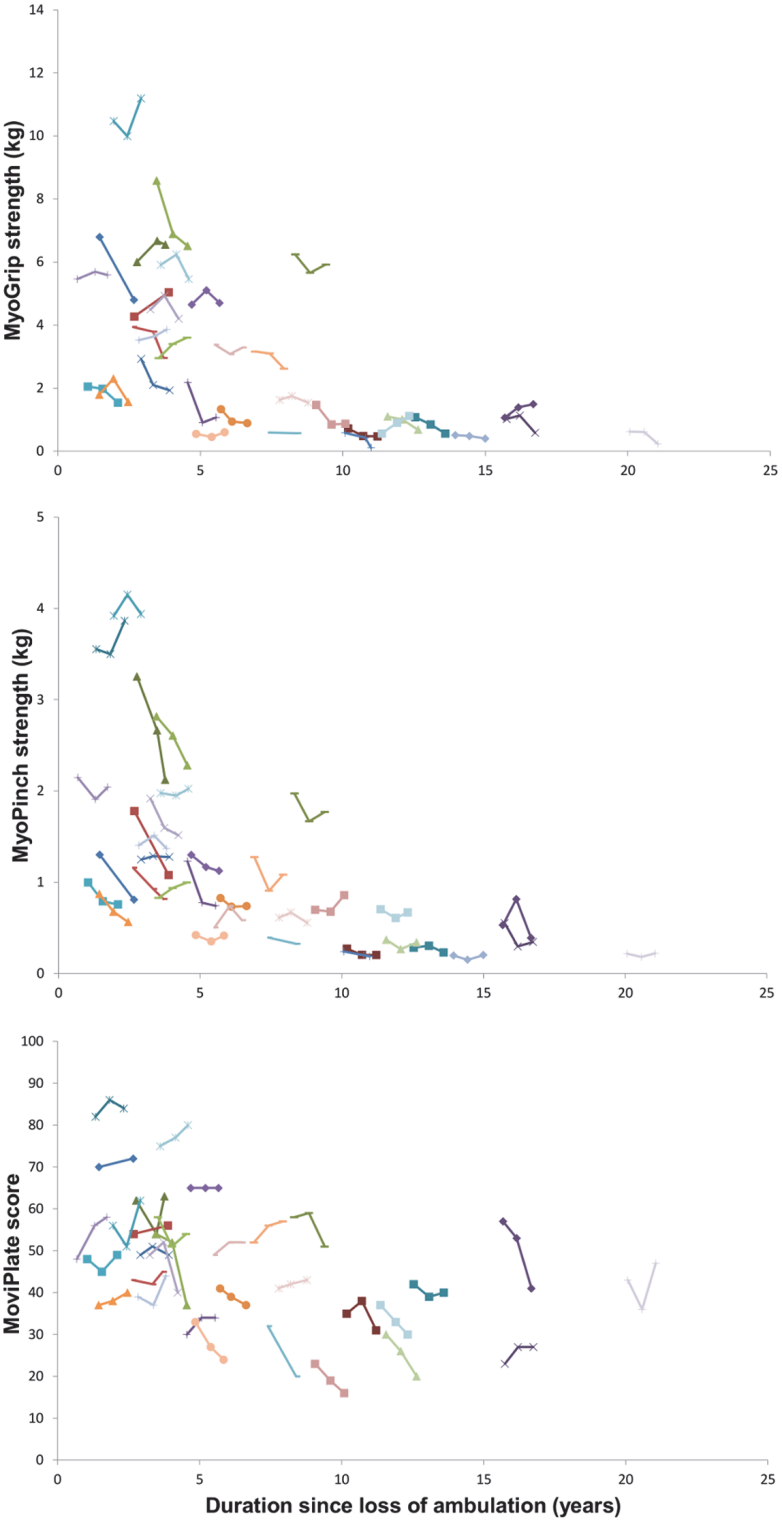
Relation between MyoSet values at baseline, at 6 months, and at one year with duration since loss of ambulation.

**Figure 4:**
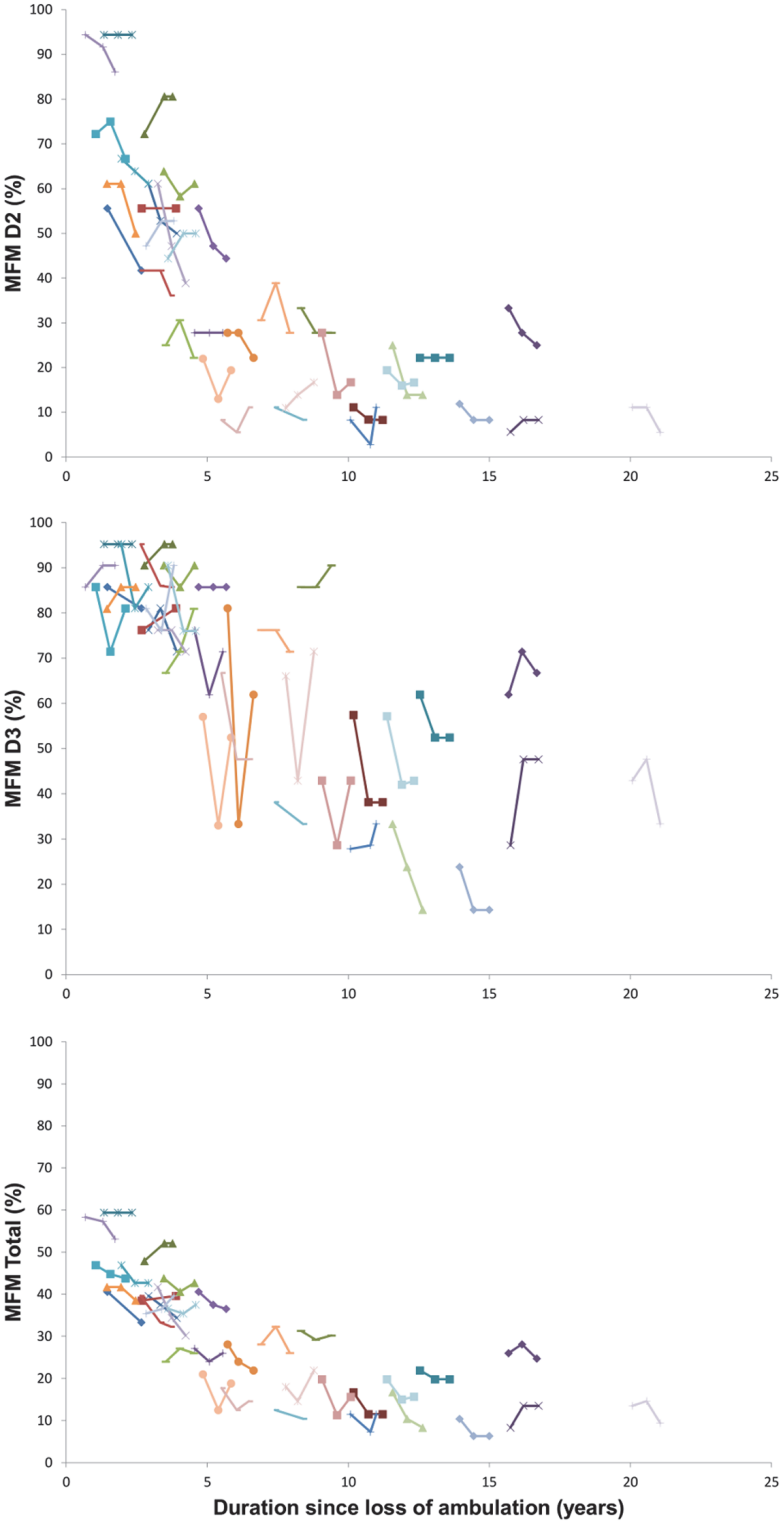
Relation between MFM-D2 and MFM-D3 sub-scores and total score and duration since loss of ambulation at baseline at 6 months and at one year.

DMD patients showed a significant decrease in distal strength but not in function from baseline to 6 months and to one year for both dominant and non-dominant sides (Fig. 3, Table 5). This decrease was observed in all DMD patients. Significant negative correlations between the MFM total score or D2 sub-score and duration since loss of ambulation were also observed (Fig. 4, Table 6).

**Table 5:** Differences in MyoSet scores between baseline and one year.

	**Non-dominant side**			**Dominant side**		
	**N**	**mean diff (SD)**	**median [min-max]**	**N**	**mean diff (SD)**	**median [min_max]**
MyoGrip (kg)	34	-0.31 (0.46) **	-0.30 [-1.41–0.54] **	34	-0.28 (0.65) *	-0.31 [-2.07–0.77] *
MyoPinch (kg)	35	-0.22 (0.31) **	-0.15 [-1.48–0.18] **	35	-0.17 (0.28) **	-0.07 [-1.13–0.31]**
MoviPlate (#)	33	-0.73 (6.24)	-1 [-15–12]	33	-1.27 (6.66)	1 [-17–10]

*p-value<0.05, **p-value<0.01

**Table 6:** Differences in MFM scores between baseline and one-year follow-up.

	**N**	**mean diff (SD)**	**median [min-max]**
MFM-D2	35	-3.50 (6.69) **	-2.80 [-22.2–8.4] **
MFM-D3	35	-3.24 (9.42)	-4.75 [-19.3–19.0]
MFM-Total	35	-2.29 (3.79) **	-2.20 [-11.5–5.2] **

*p-value<0.05, **p-value<0.01.

The changes in MoviPlate score from baseline to one year on each side were significantly correlated with the duration since loss of ambulation (rho -0.49, p<0.01 and -0.37, p <0.05 for the non-dominant hand and for the dominant hand, respectively). However, MoviPlate scores did not capture a significant change overall in the patients group over the one-year period. The evolution over the one-year period was very different for younger and older patients (Fig. 4). Patients who had spent less than 3 years in a wheelchair showed a significant increase in motor ability score as measured by MoviPlate on the dominant side. In patients who had spent 3 or more years in a wheelchair, a significant decrease of the MoviPlate score was observed on both sides (Table 7).

**Table 7:** Differences in MoviPlate scores on dominant and non-dominant sides in subjects grouped based on duration since loss of ambulation.

	**Non-dominant side**			**Dominant side**		
**Duration since loss of ambulation**	**N**	**mean diff (SD)**	**median** **[min-max]**	**N**	**mean diff (SD)**	**median** **[min-max]**
< 3 years	11	3.45 (5.72)	4 [-6–12]	11	3.09 (2.88) **	2 [0–10] **
≥ 3 years	21	-3 (5.54) *	-5 [-15–7] *	21	-3.86 (6.89) *	-4 [-17–5] *

*p-value<0.05, **p-value<0.01.

### Sample size estimation in clinical trials

We estimated the minimum number of patients who must be enrolled in a future clinical trial with outcomes measured using the MyoSet to demonstrate a significant effect of a given intervention in stabilizing the disease during one year. This estimation was performed for all patients and for patients who had spent three or more years in the wheelchair. The results are summarized in Table 8. The number of subjects required was the lowest for MyoPinch and was lower for the MoviPlate in patients who had lost ambulation less than 3 years before the trial began than for those who had been wheelchair-bound for more than 3 years.

**Table 8:** Sample size estimation per group in a clinical trial to detect an improvement stabilization of strength or MoviPlate score on the dominant (D) side.

	**Sample size/group**
MyoGrip-D (kg)	87
MyoPinch-D (kg)	44
MoviPlate-D (#)	433
≥3y-MoviPlate-D (#)	50

≥3y-MoviPlate-D—patients with duration since loss of ambulation of 3 years or more.

## Discussion

We demonstrated that the use of sensitive dynamometers could capture changes over the course of a year in non-ambulant DMD patients of all ages. The MoviPlate test only captured a significant change in patients who had been wheelchair bound for more than 3 years but not those who had lost ambulation more recently. Our study confirmed the preliminary data previously reported regarding feasibility and reliability of the MyoSet and the correlation between distal strength and clinical outcomes such as FVC, Brooke score, age, and duration since loss of ambulation [26]. We have recently demonstrated similar reliability of upper limb assessment in spinal muscular atrophy type II and type III patients, with ability to follow disease progression and loss of strength and function over a one-year period in patients older than 14 years (Seferian et al., unpublished data).

Our study population is representative of the general French DMD population. The mean age of loss of ambulation in our study population (i.e., 10 years) was similar to that observed by Desguerre *et al*. in 75 DMD patients aged from 4 to 18 years [30] and in over 300 patients recorded in the Cochin database (unpublished data), part of the UMD-DMD France database (http://www.umd.be/DMD/).

As steroid treatment is approached differently in clinical centers in France and Belgium and that only recently [2] the practice is beginning to be homogenized over the country for ambulant patients, there is little information regarding the use of steroids in non-ambulant patients. It is no wonder that only 8 patients from our study were on steroids. It is very obvious that older patients did not benefit from the same clinical and therapeutically management as the young DMD. It has already been stated that long-term steroid treatment may reduce the need for spine surgery [31]. In a retrospective study, Dooley *et al*. found that only 26% of 81 boys who had received steroid therapy had undergone spinal surgery by the age of 18, compared with 69% of boys who had not received steroid therapy by age 19 [31]. Thirty one (58.5%) out of 53 of our patients underwent spine surgery; this high rate could be explained by the scarce use of steroids. None of the patients who completed the study underwent spine surgery during the one year of evaluation. The age of spine surgery in our cohort was also consistent with the data published by Roberto *et al*. (14 years in the present paper vs. 14.2 years in 174 DMD patients with ages between 9 and 35) [32]. Despite the low rate of steroids use and the high incidence of spine surgery in older patient, this cohort is representative of the French and Belgian French-speaking DMD population in 2012 as followed in six reference centers, since there was no obvious selection bias in the recruitment.

All our DMD subjects were not enrolled in any other concomitant treatment or observational study as indicated by the good clinical practices.

One limitation of our study is related to the drop-out rate at one year of 33.9% and 6-month data are missing from 11 patients (20.7%). The fact that tests were done apart and not in relation with a regularly check-up could have impacted the lesser number of patients accomplishing the one-year visits. We initially chose to conduct assessments outside the regular yearly visit to avoid fatigue during the tests in weak patients who undergo several clinical consultation the same day during their hospital yearly visit. However, we can not rule out that the drop-out of the study was not more important in patients who experienced more rapid deterioration, or achieved significant disease milestones such as night or day ventilation, which may decrease the motivation to take part in an observational study. Similar drop-out rate at one year in observational studies has been observed by others. In a longitudinal study involving 303 patients with different neuromuscular diseases (including DMD) at baseline, the drop-out rate at one year was 46.53% [33].

One limitation of the tools used is that they assess only distal strength and function. Distal function is clinically meaningful for most of non-ambulatory patients since it reflects the ability to write, to use a mobile phone or other portable electronic devices, or to use a joystick driven wheelchair. There are only a few validated measures that assess distal upper limb strength or function. Strength can be assessed by Manual Muscle Testing or hand held myometry. Recently, a collaborative effort of a Clinical Outcome Group made up of clinicians, scientists, parent advocacy groups and industries has led to the construction of an upper limb functional scale that takes into account the proximal and distal function of upper limb [13]. Collection of longitudinal data is currently ongoing.

Another shortcoming remains in defining which differences in strength are clinically meaningful for different age groups. We showed that function is directly correlated with strength as measured by the MyoSet and MFM-D3. The MFM-D3 scores distal motricity, though the strength scores were better correlated with the MFM scores at baseline than at later times, as shown here.

Preliminary data indicate that exon skipping treatment effects are much more evident over longer periods of time than one year [34]. Therefore, sensitive devices that capture a significant change over a 6- to 12-month period would be useful outcome measures in a phase II trial or to aid in making a decision for an extension study or for treatment evaluation in a cohort of non-ambulant patients in whom a formal phase III trial is not possible to design.

DMD patients begin to experience distal upper limb weakness while still ambulant with a proximal to distal progression; grip and key pinch strengths and hand function are thus an indicator of the disease progression.

In a cross-sectional study, Mattar and Sobreira showed that hand strength in DMD patients increased during their first decade [35]. Our study was not designed to reproduce the same results as our population was slightly older compared to theirs (4.3 to 21.9 years vs. 9 to 28 years). The strength values were all correlated with the indices of severity of the disease. Indeed, the values were inversely correlated with Brooke score and FVC and with time spent in the wheelchair. It is known that a severe motor phenotype is significantly associated with lower values of FVC [36]. This correlation was stronger for the strength tests than for the MoviPlate test.

Strength tests (MyoPinch and MyoGrip) seem to be more sensitive than MoviPlate probably because strength decline is a precursor of function decline and that the relationship between both is not linear. Even with a significant decrease of strength, weak subjects can still manage compensatory mechanisms that enable them to perform well at the MoviPlate. The question that is still to be answered is whether a recuperation of strength due to a therapeutic product could have an impact on the subject function. From our point of view, it is important to record both strength and function evolution even if both do not show a significant decline over a year. Most functional assessments with existing methods (scales) do not reflect common disease stages. Even the weakest patients scored below the sensitivity threshold of traditional dynamometers.

Longitudinal data on the sensitivity to change of outcome measures in non-ambulant DMD patients are critically lacking, and this is one of the main reasons why clinical trials are not conducted in this population. A recent study of 41 ambulant and non-ambulant DMD patients showed a good responsiveness (ability to change or stability in absence of change) of the MFM score and sub-scores [33]. The 12-month standardized mean score (individual scores were transformed into annual score changes by linear interpolation) changes reported by Vuillerot *et al*. showed higher negative values (-7.5 for MFM D2, -4.2 for MFM D3, and -5.8 for the total score, all p<0.05) than our one-year mean scores (-3.5, -3.2 and -2.3, respectively). The differences are due to the fact the Vuillerot et al. analysis included both ambulant and non-ambulant DMD patients (10 or the 41 were ambulant). Although the population described by Vuillerot *et al*. is younger than our population (mean age 13 vs. 17.9) and had clinically less severe disease (mean Brooke score 3.3 vs. 4.7), these patients appeared to be declining more rapidly than our study group. One explanation could be that our older subjects have more stable disease. Another possibility is related to a limitation of our study: that is the patients who were not evaluated at 1 year (18/53) may constitute a sub-group with a more severe decline.

MoviPlate does not appear to be a sensible tool for use in early non-ambulant patients. It may be of interest as a potential outcome measure in patients who have spent more than 3 years in the wheelchair, however. As the disease progresses, wrist and long finger flexors develop contractures that could limit the utility of this tool. However, these patients seem to find compensating strategies when performing on the MoviPlate, as described in the method section. These different strategies for the MoviPlate test must be taken into consideration when interpreting the data and could explain the lack of correlation at one year for all patients. The significant increase in the MoviPlate score for patients wheelchair bound for less than 3 years was not reflected in improvements in the distal strength tests. This is probably related to motor learning effect in a test where motor strategy certainly plays a role in the strongest patients. In the weakest, strength probably becomes the limiting factor. There is also probably a ceiling effect above which strength decrement does not impact the functional test as it does below this threshold.

Based on our data, we were able to calculate the sample size required to detect significant effects using MyoSet devices in a placebo-controlled therapeutic trial of a compound expected to stabilize disease progression or improve motor function for wheel-chair bound patients. We determined that about 50 patients/group are necessary to detect a significant effect for MoviPlate (for patients with duration since loss of ambulation of 3 years or more) and MyoPinch. This number of patients is similar to numbers of subjects in trials ongoing in the DMD field; ongoing phase III trials of tadalafil (Lilly) and ataluren (PTC therapeutics) include more than 200 patients. Our data indicated that these tools should be considered for trials conducted in non-ambulant patients but that time spent in the wheelchair should be considered. Beside the feasibility in term of number of patients, one must consider that a clinical trial in non-ambulant patients requires tests that are sensitive enough to capture a change during the one year period of the trial. Even if the change measured during this period of time falls below the estimated clinically significant change, it may constitute a strong evidence that further follow up of these patients is required, before concluding that the given drug has no effect in the considered population. Comparison with other outcome measures, such as the PUL module (14, 21) require recording in the same population, as it will be the case in the SKIP-NMD trial for exon skipping 53 (Sarepta). Given the demonstration of feasibility, high reliability, and sensitivity to negative change at one year as well as good correlation with other clinically relevant variables, these innovative measures represent a very promising approach to asses a therapeutic intervention that aims to maintain muscle strength and function in non-ambulant DMD patients.

## Supporting Information

S1 Protocol Trial Protocol (French). (PDF)

S2 Protocol Trial Protocol (English). (PDF)

S1 TREND Checklist TREND Checklist. (PDF)
